# Safety of direct oral anticoagulants in patients with hereditary hemorrhagic telangiectasia

**DOI:** 10.1186/s13023-019-1179-1

**Published:** 2019-08-28

**Authors:** C. L. Shovlin, C. M. Millar, F. Droege, A. Kjeldsen, G. Manfredi, P. Suppressa, S. Ugolini, N. Coote, A. D. Fialla, U. Geisthoff, G. M. Lenato, H. J. Mager, F. Pagella, M. C. Post, C. Sabbà, U. Sure, P. M. Torring, S. Dupuis-Girod, E. Buscarini, VASCERN-HHT

**Affiliations:** 1 VASCERN HHT Reference Centre, Hammersmith Hospital, Imperial College Healthcare National Health Service Trust, London, and Imperial College London, London, UK; 2 EuroBloodNet Reference Centre and Centre for Haematology, Imperial College Academic Health Sciences Centre, Imperial College London, London, UK; 3 VASCERN HHT Reference Centre, Essen University Hospital, Departments of Otorhinolaryngology and Neurosurgery, University of Duisburg-Essen, Essen, Germany; 4 VASCERN HHT Reference Centre, Odense Universitetshospital, Syddansk Universitet, Odense, Denmark; 5 VASCERN HHT Reference Centre, ASST Maggiore Hospital, Crema, Italy; 6 VASCERN HHT Reference Centre, Centro sovraziendale Malattie rare, Frugoni Internal Medicine Unit - University of Bari A Moro, Bari, Italy; 7 VASCERN HHT Reference Centre, Department of Otorhinolaryngology, University of Pavia, IRCCS Policlinico San Matteo Foundation, Pavia, Italy; 8 VASCERN HHT Reference Centre, St Antonius Ziekenhuis, Nieuwegein, Netherlands; 9 VASCERN HHT Reference Centre, Genetic department, Hospices Civils de Lyon, Femme-Mère-Enfants Hospital, F-69677 Bron, France

**Keywords:** Apixaban, Atrial fibrillation, Dabigatran, Epistaxis, Heparin, Pulmonary emboli, Rivaroxaban, Venous thromboemboli, Warfarin

## Abstract

**Background:**

Hereditary hemorrhagic telangiectasia (HHT) is a rare vascular dysplasia resulting in visceral arteriovenous malformations and smaller mucocutaneous telangiectasia. Most patients experience recurrent nosebleeds and become anemic without iron supplementation. However, thousands may require anticoagulation for conditions such as venous thromboembolism and/or atrial fibrillation. Over decades, tolerance data has been published for almost 200 HHT-affected users of warfarin and heparins, but there are no published data for the newer direct oral anticoagulants (DOACs) in HHT.

**Methods:**

To provide such data, a retrospective audit was conducted across the eight HHT centres of the European Reference Network for Rare Multisystemic Vascular Diseases (VASCERN), in Denmark, France, Germany, Italy, the Netherlands and the UK.

**Results:**

Although HHT Centres had not specifically recommended the use of DOACs, 32 treatment episodes had been initiated by other clinicians in 28 patients reviewed at the Centres, at median age 65 years (range 30–84). Indications were for atrial fibrillation (16 treatment episodes) and venous thromboembolism (16 episodes). The 32 treatment episodes used Apixaban (*n* = 15), Rivaroxaban (*n* = 14), and Dabigatran (*n* = 3). HHT nosebleeds increased in severity in 24/32 treatment episodes (75%), leading to treatment discontinuation in 11 (34.4%). Treatment discontinuation was required for 4/15 (26.7%) Apixaban episodes and 7/14 (50%) Rivaroxaban episodes. By a 4 point scale of increasing severity, there was a trend for Rivaroxaban to be associated with a greater bleeding risk both including and excluding patients who had used more than one agent (age-adjusted coefficients 0.61 (95% confidence intervals 0.11, 1.20) and 0.74 (95% confidence intervals 0.12, 1.36) respectively. Associations were maintained after adjustment for gender and treatment indication. Extreme hemorrhagic responses, worse than anything experienced previously, with individual nosebleeds lasting hours requiring hospital admissions, blood transfusions and in all cases treatment discontinuation, occurred in 5/14 (35.7%) Rivaroxaban episodes compared to 3/15 (20%) Apixaban episodes and published rates of ~ 5% for warfarin and heparin.

**Conclusions:**

Currently, conventional heparin and warfarin remain first choice anticoagulants in HHT. If newer anticoagulants are considered, although study numbers are small, at this stage Apixaban appears to be associated with lesser bleeding risk than Rivaroxaban.

**Electronic supplementary material:**

The online version of this article (10.1186/s13023-019-1179-1) contains supplementary material, which is available to authorized users.

## Background

Hereditary hemorrhagic telangiectasia (HHT) is an inherited disorder of angiogenesis affecting one in 6,000 individuals [1, 2]. The most common clinical signs include recurrent epistaxis (nosebleeds), and visible telangiectases on the skin, mucous membranes, and in the gastrointestinal tract. Imaging modalities such as computerised tomography, magnetic resonance imaging (MRI) and ultrasound commonly detect visceral arteriovenous malformations (AVMs). Current estimates are that pulmonary AVMs affect approximately 50% of patients, hepatic AVMs approximately 50%, with other organs such as the brain, gastrointestinal tract and pancreas affected less frequently. AVMs lead to organ-specific issues discussed in detail elsewhere [2].

Bleeding and anemia are major foci in clinical management of HHT patients [3]. They are also established priorities for the HHT patient/professional consortium of the European Reference Network for Rare Multisystemic Vascular Diseases (VASCERN [4]) - management of nosebleeds, and assessment of iron deficiency, are two of the five HHT Outcome Measures defined by VASCERN HHT [2]. Compared to recurrent bleeds from nasal or gastrointestinal telangiectasia in HHT, hemorrhage from visceral AVMs is less frequent, but can be life-changing or life-limiting, for example hemorrhagic strokes from cerebral AVMs [5], and hemoptysis or hemothorax from pulmonary AVMs [6]. It is not possible to exclude all HHT visceral involvement by imaging, since abnormal vessels may be below the resolution of conventional techniques, and identifiable only by organ inspection at surgery, or histology [7, 8]. The frequency of small vessel involvement at internal sites in HHT is difficult to ascertain, and likely important since high proportions of even the smallest skin telangiectasia have been shown to contain direct arteriovenous communications [8]. As a result, in high risk situations such as pregnancy, management of an individual with HHT often proceeds assuming all potential sites are affected [9].

The current manuscript focuses on anticoagulation in HHT. Not infrequently, HHT patients develop conditions where anticoagulants would usually be prescribed, particularly venous thromboembolism (VTE [10]), and atrial fibrillation (AF, [11–13]). Using published VTE and atrial fibrillation rates in HHT [10–13], the number of HHT affected individuals likely to develop an indication for anticoagulant treatment can be estimated to be up to 10,000 cases in Europe, and 6000 in North America. Almost all of these treatments will be initiated in emergency and acute settings, where experience with HHT is likely to be limited.

Anticoagulants may be used with caution in selected patients with a bleeding disorder [14]. Unbiased data capture methods indicate reasonable tolerance of heparin and warfarin by HHT patients, although hemorrhage was a recognised adverse event across 148 cases spanning oral warfarin (*n* = 55), unfractionated heparin (*n* = 41) and subcutaneously administered heparin (low molecular weight or unfractionated, *n* = 52) [15]. As a result, in striking a balance between potential bleeding and life-threatening thrombotic risks, where prophylactic or treatment dose anticoagulation would be indicated for a member of the general population without HHT, anticoagulation is usually cautiously recommended for patients with HHT, and efforts made to find the anticoagulant that is best suited to the individual [16].

Four new direct oral anticoagulants (DOACs) have become available in recent years and are now widely used in the general population in the settings of non-valvular atrial fibrillation (AF) and venous thromboembolism (VTE). Three of these agents directly inhibit factor Xa (FXa) - Rivaroxaban (Xarelto, Bayer), Apixaban (Eliquis, (Pfizer/ Bristol-Myers Squibb) and Edoxaban (Lixiana/ Daiichi Sankyo), while Dabigatran (Pradaxa, Boehringer Ingelheim) is a direct thrombin inhibitor. Within their licensed indications, DOACs have been shown to be at least as efficacious as warfarin and demonstrate some clear safety benefits over warfarin, in particular reducing the risk of intracranial haemorrhage. DOACs offer significant advantages to patients and doctors, and careful selection for the relevant indication can simplify therapy while improving outcomes in the general population [17]. Indeed, general European guidance recommends the use of DOACs over warfarin for stroke prevention in patients with nonvalvular AF [18], while UK NICE guidance recommends that the decision between DOAC and warfarin be based on the patient’s clinical features and preferences [19].

While bleeding is a recognised complication of DOACs in the general population, the incidence of intracranial and other significant major bleeding has generally been shown to be lower than for warfarin [17]. An important exception to this, and pertinent to the HHT population, is gastrointestinal bleeding, the incidence of which appears to vary considerably between DOACs. Higher rates of gastrointestinal bleeding in DOACs compared to warfarin were demonstrated in some of the large AF clinical trials [20–22] and post marketing studies [23, 24], however this was not found in the VTE trials, possibly reflecting a younger population with fewer co-morbidities. The risks of gastrointestinal bleeding or underlying gastrointestinal pathology are important considerations when selecting a DOAC.

Whether the degree of DOAC-related bleeding would be more or less severe in HHT patients compared to the general population, or compared to HHT patients using warfarin or heparin [15], was not known. Similarly, the likely requirement for reversibility of DOAC activity in HHT patients was not known, and this is particularly pertinent since reversal agents are not yet available for all DOACs. No specific trials have been undertaken in HHT, nor are they likely to be undertaken for this subgroup of patients with an already rare disease.

The importance of acquiring drug tolerability data for patients with rare diseases has been emphasised by the European Commission. In particular, the fostering of information on safety standards for rare disease patients was one of the core recommendations of the auditing authority for the European Reference Networks (ERNs) for Rare Diseases which were launched in 2016. This focus on safety standards was adopted by the ERN for Rare Multisystemic Vascular Diseases (VASCERN) [3]*,* which includes a working group dedicated to HHT.

As for anti-angiogenic agents in HHT [25], the aim of the current study was to evaluate the use, safety and tolerability of novel anticoagulant agents in HHT patients treated within HHT expert Centers across the virtual network of VASCERN.

## Results

### HHT Reference Centre prescriptions

None of the eight VASCERN HHT Reference Centres in Denmark, France, Germany, Italy, the Netherlands or the UK reported prescribing DOACs or a new anticoagulant to patients with HHT. Therefore no patients could be entered into a prospective study.

### Demographics of cohort identified by Centres

Seven of the VASCERN HHT Reference Centres reported that they had reviewed HHT patients treated with DOACs. Treatments had been initiated by diverse external institutions as part of conventional general population emergency and acute care management protocols. These cases were systematically audited and analysed as a retrospective study.

Individual cases are presented in Additional file 1: Table S1. Briefly, 32 treatment episodes were identified in 28 cases. All 28 patients had a least 3 Curaçao Criteria with known visceral involvement including hepatic AVMs (*n* = 15), pulmonary AVMs (*n* = 6), and cerebral AVMs (*n* = 3). Twenty of the 28 cases had a known HHT genotype- 6 *ENG*, 14 *ACVRL1.* Ages ranged from 30 to 84 (median 65) years. None were known to have renal insufficiency.

As indicated in Table 1, anticoagulation indications were for atrial fibrillation (AF, 13 cases, 16 treatment episodes), or VTE (15 cases, 16 treatment episodes). The AF patients were older (mean 69.9 ys (range 53–84 years) vs 56.1 (range 30–75 years, *p* = 0.0061), more likely to be male (76.9% of 13 vs 20.0% of 15, *p* = 0.0031), and less likely to have pulmonary AVMs than the VTE patients (Table 1).

**Table 1:** Patient Demographics

	Total Cases (*N* = 28)	VTE Cases (*N* = 15)	AF cases (*N* = 13)	*P* value €
Cases per Centre, range (median)	0–12 (2)	0–7 (1)	0–5 (2)	–
Age, years, range (median)	30–84 (65)	30–75 (57)	53–84 (71)	0.0061
Males, N (%)	13 (46.4%)	3 (20%)	10 (76.9%)	0.0031
Pulmonary AVMs (%)	6 (21.4%)	6 (40%)	0 (0%)	0.010
Hepatic AVMs (%)†	15 (75%)	5/8 known (63%)	10/12 known (83.3%)	0.30
Cerebral AVMs (%) ‡	3 (16.7%)	2/10 known (20%)	1/8 known (12.5%)	0.68
DOAC episodes per patient, range (median)	1–3 (1)	1–3 (1)	1–1 (1)	–
DOAC cases also treated with warfarin	7	3	4	–

Demographics of the 28 cases: Of the 15 VTE cases, 10 required treatment for pulmonary thromboemboli (+/− deep venous thromboses), 3 had apparently isolated lower limb deep venous thromboses, one had a mesenteric artery thrombosis, and one a thrombosis in a peripherally inserted central venous catheter. † 20 cases screened. ‡ 18 cases screened. € *p* values calculated by the Chi squared test, except for age which was calculated by Mann Whitney

### Treatment demographics

15 cases were treated with Apixaban, 14 with Rivaroxaban, (2 with both); and 3 with Dabigatran (1 after Apixaban), all using conventional dosing schedules (Additional file 2: Table S2).

There was no difference in age, gender, HHT status, genotype, country/healthcare provider, or indication between patients treated with Apixaban, Rivaroxaban or Dabigatran (Table 2).

**Table 2:** Treatment Agent Comparisons

	All Agents	Apixaban (*N* = 15)	Rivaroxaban (*N* = 14)	Dabigatran (*N* = 3)	*P* value €
Age, years	30–84 (median 65)	31–80 (median 67)	30–84 (median 60)	57–77 (median 66)	0.64
Gender	16 male, 16 female	7 male, 8 female	8 male, 6 female	1 male, 2 female	0.71
AF indication, N (%)	16 (50%)	6 (43%)	9 (60%)	1 (33%)	0.59
VTE indication, N (%)	16 (50%)	9 (57%)	5 (40%)	2 (66%)	0.59
Nosebleeds improved, N	1	0	0	1 }	0.044
Nosebleeds unchanged, N	7	4	2	1 }	
Worse but tolerable, N	13	7†	5	1 }	
Worse, treatment discontinued, N	11 ‡	4	7	0 }	

Treatment Agent Comparisons: † In one case only achieved after halving of the treatment dose. € p values calculated by the Chi squared test, except for age which was calculated by Kruskal Wallis. ‡ For the eleven episodes where DOAC treatment needed to be discontinued, subsequent management is described in the text and summarised in Additional file 1: Table S1. There was no difference between the 3 agents in recipient HHT status, genotype, or country/healthcare provider (data not shown)

### Nosebleeds and DOAC treatments

Across the 32 treatment episodes, nosebleeds were unchanged in 7 (21.9%). One treatment episode was reported to improve nosebleeds. 24 episodes (75.0%) were associated with a reported increase in nosebleed severity. In 11 episodes (34.4%), this led to treatment discontinuation (Table 2), in a further case to a reduced (half) dose, and in 9 cases directly to referral to the VASCERN HHT Reference Centre.

To correct for any potential ascertainment bias, the 23 episodes that had not precipitated VASCERN reference centre referral were examined. These showed similar findings to the full dataset: nosebleeds were improved in one (4.4%), unchanged in 7 (30.4%); increased in 9 (39.1%); and led to treatment discontinuation in 6 (26.1%).

There was no difference in reported nosebleed changes according to age, gender, known HHT genotype, pulmonary, hepatic, or cerebral AVM status; treatment indication (AF vs VTE), or the country/healthcare provider reporting (*p* > 0.21).

### Agent-specific analyses

As illustrated in Table 2, overall, the proportion of episodes accompanied by nosebleed worsening was 11/15 (73.3%) for Apixaban, 12/14 (85.7%) for Rivaroxaban, and 1/3 (33.3%) for Dabigatran, with one Dabigatran user describing nosebleed improvement. Increases in nosebleed severity meant that 7 of 14 (50%) Rivaroxaban users had to discontinue treatment, compared to 4 of 15 using Apixaban (26.7%), and no users of Dabigatran (Table 2).

A four point scale (improved, unchanged, worse but tolerable, and worse leading to treatment discontinuation) across the three agents in the 32 episodes suggested differences between the agents, with Rivaroxaban displaying more cases in the more severe categories (Table 2, chi squared *p* = 0.044).

To examine if the apparent difference in nosebleed responses may be due to chance distributions of other differences between the patients, multiple regression analyses were performed. The distribution of the 4 reported nosebleed changes was considered sufficiently normal (Additional file 3: Figure S1), to be used as the dependent variable. The crude trend for Rivaroxaban to be associated with increased bleed severity was maintained after adjustment for gender, and strengthened after adjustment for age, treatment indication, or all 3 variables (coefficients and *p* values presented in Table 3).

**Table 3:** Relationship between Rivaroxaban and bleed severity calculated by multiple regression

Adjusted for	Coefficient for Rivaroxaban (95% confidence intervals)	Adjusted r squared for model	*P* value
Nil (crude regression)	0.67 (0.04, 1.30)	0.14	0.038
Sex	0.67 (0.024, 1.31)	0.17	0.043
Age	0.74 (0.12, 1.36)	0.18	0.021
Indication (AF or VTE)	0.79 (0.18, 1.41)	0.22	0.014
Age and indication	0.81 (0.18, 1.43)	0.22	0.014
Age, sex and indication	0.89 (0.26, 1.51)	0.24	0.008

Linear regression of Rivaroxaban against outcome variable of the 4 point nosebleed scale (improved, unchanged, worse but tolerated, worse leading to treatment discontinuation) across the 25 patients with a single treatment episode, compared to the other two agents (11 Rivaroxaban-users vs. 14 non-Rivaroxaban-users, either Apixaban or Dabigatran). There were similar relationships when including the three patients who had used more than one DOAC (data not shown)

One individual using Rivaroxaban also reported worsening of gastrointestinal bleeding in addition to epistaxis. None of the three premenopausal women reported increased menstrual bleeding with Rivaroxaban (*n* = 2) or Apixaban (*n* = 1): in two of these individuals there was either minimal or no reported worsening of nosebleeds, while in the third, treatment was rapidly discontinued due to nosebleeds.

### Alternate anticoagulants

Of the 11 individuals unable to tolerate a DOAC due to nosebleed exacerbation, 3 were successfully able to use an alternate DOAC (Apixaban, Rivaroxaban, or Dabigatran). Five of 7 treated with warfarin before or after DOAC treatment were able to tolerate the conventional anticoagulant, noting nosebleeds were reported to be increased in two of these patients (Additional file 1: Table S1).

Four patients who discontinued Rivaroxaban or Apixaban due to excessive nosebleeds were started on warfarin: two tolerated warfarin (for at least 3 months), two did not, but of these, one was able to tolerate heparin, and one was able to tolerate Apixaban (Additional file 1: Table S1). In addition to this patient who discontinued Rivaroxaban due to excessive nosebleeds and was able to tolerate Apixaban, one patient who discontinued Apixaban due to excessive nosebleeds was able to tolerate Rivaroxaban, and a second was able to tolerate Dabigatran (Additional file 1: Table S1). One patient had two treatment periods 3 years apart and was able to tolerate Rivaroxaban and warfarin without problems on the first occasion, but had more severe nosebleeds on both Rivaroxaban and Apixaban 3 years later (Additional file 1: Table S1).

In 5 patients who discontinued Rivaroxaban or Apixaban due to excessive nosebleeds, no further anticoagulant was used, for AF (*n* = 3), and VTE (*n* = 2). Two of the AF patients had left atrial appendage occlusions performed.

### Qualitative comparison to conventional anticoagulant agents

Eight DOAC users reported nosebleeds worse than anything experienced previously, with individual arterial-like nosebleeds lasting several hours requiring hospital admissions, blood transfusions, and in all cases leading to treatment discontinuation. Details of the precise phrases are provided in the Additional file 1: Table S1. These extreme responses were reported by 3 of 15 (20%) Apixaban users and 5 of 14 (35.7%) Rivaroxaban users.

Data from the current series were compared to previously published data in HHT patients treated with heparin or warfarin ([15], Table 4). Noting that study populations and assessments were not fully comparable, bleeding rates, including extreme (i.e. worse than anything experienced previously), appeared to be higher in DOAC-treated HHT patients (Table 4).

**Table 4:** Comparison with conventional anticoagulants

	Total of HHT cases	Nosebleeds no different or better	Nosebleeds Worse- all severities	Nosebleeds Worse- extreme responses
Warfarin	64	22 (34.3%)^†^	39 (60.9%)	3 (4.7%) ^‡^
Subcutaneous heparin^	52	22 (42.3%) ^†^	28 (53.9 [39.8, 67.9])	None recorded^‡^
Intravenous heparin	41	16 (39.0%) ^†^	22 (53.7 [37.7, 69.6])	2 (4.9%)^‡^
Apixaban	15	4 (26.7%)	11 (73.3%)	3 (20%)
Rivaroxaban	14	2 (14.3%)	12 (85.7%)	5 (35.7%)
Dabigatran	5 ^€^	2 (40%) ^†^	3 (60%)	None recorded ^‡^

Comparison of current series and data in different study populations previously published in [15]. ^†^Improvement in nosebleeds were reported for warfarin (*n* = 2), subcutaneous heparin (*n* = 2), intravenous heparin (*n* = 3) and Dabigatran (*n* = 1). ^‡^ Extended analysis of comments in primary SurveyMonkey data. ^ includes low molecular weight heparins. ^€^Includes 2 published cases [15] with dosages of 75 mg a day. While the different methodologies and study populations mean that any comparative statistics should be used with caution, the Chi-squared p values comparing two categories (not worse/worse) to warfarin were Apixaban *p* = 0.56; Rivaroxaban *p* = 0.20. Chi-squared *p* values comparing 3 categories (not worse, worse but not extreme, extreme) to warfarin were Apixaban *p* = 0.15; Rivaroxaban *p* = 0.003

## Discussion

This small, retrospective study provides an important evaluation of commonly available anticoagulants in a rare disease population at increased risk of hemorrhage. In HHT, the most evident hemorrhagic risks are of mucosal origin, namely nosebleeds, which are often frequent and severe in the absence of anticoagulation [26]. Gastrointestinal bleeds from HHT telangiectases are less frequent, although bleeds from other visceral AVMs may also occur. It is important to attempt to strike a balance between the life-threatening risk of clot extension and embolisation if anticoagulation is withheld or underdosed, and the risk of bleeding that may be more amenable to medical or surgical management.

In HHT, there is a reasonable body of experience with heparin and warfarin for a rare, predominantly hemorrhagic disease [15, 27], Expert HHT Centres recommend that HHT patients should receive prophylactic anticoagulation at high risk times, as for the general population [9, 15, 16]. Trials of full anticoagulation are also generally recommended by expert Centres, noting that additional ENT treatments may be required to enable patients to tolerate anticoagulation [16]. This contrasts with management outside of expert Centres: as published in [15], 381 of 700 (54.4%) surveyed HHT patients reported that they had been advised by a doctor not to use anticoagulants because they had HHT, AVMs, or nosebleeds. Additionally, in the current study, one of the cases developed severe multiple pulmonary emboli several months after local clinicians had decided not to treat a clinically evident, ultrasound-confirmed deep vein thrombosis because of the presence of HHT and nosebleeds.

To date, anticoagulants recommended by the VASCERN HHT Centres have been restricted to warfarin and heparin for which there is published data of tolerance through non biased survey methodology [15]. With the exception of two Dabigatran cases reporting worsening nosebleeds [15], we could find no data on the tolerance of DOACs in HHT. The current series provides data on three agents, two that directly inhibit FXa (Rivaroxaban and Apixaban), and one direct thrombin inhibitor (Dabigatran). During the study recruitment period, no HHT patients using a third direct FXa inhibitor (Edoxaban) were reported, although one case was reviewed in the final stages of manuscript revision, and notably this individual with *ENG* HHT and daily, small nosebleeds, tolerated Apixaban and Edoxaban equally well, and had also previously tolerated more than 4 years of warfarin therapy for recurrent VTE.

The main study numbers are small, but importantly, are in keeping with findings in some larger studies in the general population without HHT: To date, no direct comparison is available between Apixaban and Rivaroxaban in clinical practice, therefore any difference in bleeding in randomized and non-randomized studies may be accounted for by differences in the enrolled populations. That said, it is of potential interest that compared to warfarin, gastrointestinal bleed rates are similar for Apixaban, but higher for Rivaroxaban (and Dabigatran) [20, 21, 24, 28]. Our study was unable to evaluate effects on menstrual bleeding, although notably, in the general population, menstrual bleeding has been shown to be more severe in members of the general population treated with Rivaroxaban than Apixaban [29, 30]. Reasons for the observed differences in bleeding between direct FXa inhibitors are not clear: it is possible that these may in part reflect differences in P-glycoprotein transportation and peak anticoagulant concentration, which themselves are dependent on metabolism and dosing regimen.

If a newer anticoagulant agent is under consideration in HHT, the current trends for bleeding risk would seem to favour Apixaban as a first choice, since we have shown this to have been successfully used in HHT with no dose adaptations and, with a similar HHT bleeding profile to existing anticoagulants. Although study numbers are small and may not be replicated in larger series, at this stage Apixaban also seems to be less associated with significant mucosal haemorrhage in HHT patients than Rivaroxaban. However, a reversal agent for Apixaban is unlikely to be as widely available as Vitamin K is for Warfarin. (Prothrombin complex concentrates should be administered in extreme hemorrhage due to more rapid effectiveness.)

## Conclusions

At this interim stage, we conclude that

- The oral anticoagulant of choice in HHT is warfarin, due to its tolerance data and a reversal agent that is widely available;
- Of the new direct oral anticoagulants, Apixaban appears to be associated with lesser bleeding complications/risk than Rivaroxaban;
- If an HHT patient suffers excessive nosebleeds with one particular anticoagulant, they may successfully switch to an alternate agent, though it is not currently possible to predict which agent will best suit an individual.

We believe these interim conclusions to be an appropriate and necessary measure to safeguard HHT patients, but recognise the need for further study. Prospective evaluation of future cases in a Drug Registry is recommended, and VASCERN HHT will be seeking funds to enable this.

## Methods

### Study initiation and design

The topic was introduced as potentially suitable for further study at an October 2017 Face to Face meeting of VASCERN HHT. Although most VASCERN HHT Centres reported that they had seen HHT patients with significant bleeding apparently caused by NOACs, whether the degree of such bleeding was more severe than that observed for HHT patients on heparin or warfarin was not clear.

Between January to March 2018, an audit was conducted of DOAC-treated HHT cases prospectively accrued at one VASCERN HHT Reference Centre. These results, suggesting bleeding may be more severe in certain cases, were reported at the VASCERN HHT telecon on 27 April 2018**.** It was agreed that more cases should be gathered by other Reference Centers. The need to avoid bias was discussed in relation to a) whether patients had been referred to the Centre because of a bleed (particularly true for centres led by Rhinology services), and b) differential use between countries of concurrent heparin administration. An agreed data capture proforma was developed including a report on the response post DOAC, whether nosebleeds were reported to be improved, unchanged or worse (sub-classified into tolerable, sufficiently severe to lead to treatment cessation, and extreme, unlike anything experienced previously).

The initial single centre data were shared anonymously with a manufacturer (Bayer) in May 2018. Bayer encouraged submission of the case series manuscript, and reported the adverse events to their pharmacovigilence team.

All eight VASCERN HHT Centres then reviewed their databases for HHT patients using DOACs. Early cases were presented at a Face to Face Meeting in May 2018, and data entered into the proformas for compilation. All Reference Centres continued to accrue cases. Interim analyses were discussed at a Face to Face Meeting in March 2019 in the presence of Consultant Hematologists from EuroBloodNet. The final sets of cases were submitted up to the close of data capture on 18th April 2019.

### Data analysis

For numeric analyses, reported nosebleed responses on DOAC treatments were placed in one of four categories- (1) improved; (2) unchanged; (3) worse but patient able to continue DOAC treatment, or (4) worse leading to DOAC treatment discontinuation. Qualitative analysis also recorded the description of changes in nosebleed severity.

For data analysis and to generate graphs, Excel chart data were uploaded to STATA IC v15 (Statacorp, Texas) and Graph Pad Prism 7.03. Summary statistics were generated. For three or more groups, *p* values were calculated using Kruskal Wallis with Dunn’s post test correction used. For two groups, p values were calculated by Mann Whitney, or for categorical analyses, using the Chi squared test. STATA IC v15 was also used to perform linear regression analyses.

## Additional files

Additional file 1: **Table S1.** Details of individual patients and treatments. (PDF 403 kb)

Additional file 2: **Table S2.** Dosing regimes. (PDF 279 kb)

Additional file 3: **Figure S1.** Normal quantile plot of 4 point bleeding scale. (PDF 177 kb)

## Data Availability

The datasets analysed during the current study are available from the corresponding author on reasonable request.

## Abbreviations

*ACVRL1*: Gene encoding the ALK-1 protein
AF: Atrial fibrillation
AVM(s): Arteriovenous malformation(s)
DOAC: Direct oral anticoagulant
*ENG*: Gene encoding the endoglin protein
ERNs: European Reference Networks
FXa: Factor Xa
HHT: Hereditary haemorrhagic telangiectasia
NICE: The National Institute for Health and Care Excellence
PAVMs: Pulmonary arteriovenous malformations
PE: Pulmonary emboli
VASCERN: European Reference Network for Rare Vascular Diseases
VTE: Venous thromboemboli
