# Supplementary material for: Safety of direct oral anticoagulants in patients with hereditary hemorrhagic telangiectasia
Source: Orphanet J Rare Dis. 2019 Aug 28;14:210. doi: 10.1186/s13023-019-1179-1 (PMC6714298; doi:10.1186/s13023-019-1179-1)
Supplement: Supplementary file 1 — Table S1. Details of individual patients and treatments. (PDF 403 kb) [file 13023_2019_1179_MOESM1_ESM.pdf]

Safety of direct oral anticoagulants in patients with hereditary hemorrhagic telangiectasia

Shovlin CL, Millar CM, Droege F, Kjeldsen AD, Manfredi G, Suppressa P, Ugolini S, Coote N, Fialla AD, Geisthoff U, Lenato GM, Mager HJ, Pagella F, Post MC, Sabbà C, Sure U, Torring PM, Dupuis-Girod S, Buscarini E, VASCERN-HHT.

Orphanet Journal of Rare Diseases 2019

ADDITIONAL FILE 1: Table S1: Details of individual patients and treatments

Table S1: Details of individual patients and treatments

Centre	Case	Gender	Age	Indication	Episode	Agent	Comment	Scale
1	#1	M	65	AF	1	Rivaroxaban #1	"Substantially increased nosebleeds. Previously 1 a month. 1st 2 days minor nosebleeds, then 3rd day "catastrophic" pourer/gusher lasting over 1 hr. Needed to go to hospital for emergency pack and kept in hospital for 2 days”[a]	6
					2	Warfarin	“Fine, no major bleeds in 17 months”	w
1	#2	M	76	AF	1	Rivaroxaban #2	“Fountains of nosebleeds every day”	6
					2	Warfarin	“Still bled”	w
					3	Apixaban #1	“"1 every 1-2/52, trivial"	2
1	#3	F	30	VTE	1	Rivaroxaban #3	“Nose bleeds increased to 4 times a day”	3
					2	Warfarin	“Usual nosebleeds, no more than one a day”	w
1	#4	F	71	VTE	1	Rivaroxaban #4	"Room like a slaughter house". Hours-long nosebleeds every 3d. Nosebleeds settled 1 month after stopping. [b].	6
1	#5	F	39	VTE	1	Rivaroxaban #5 [c]	Local doctors contacted us for advice - "intolerant due to nosebleeds”	5
1	#6	M	73	AF	1	Apixaban #2	“Nosebleeds worse. Pourers lasting many hours. Daily shorter bleeds. Needed more intravenous iron” ‡	3
1	#7	F	77	AF	1	Apixaban #3	"Torrential" posterior pharyngeal bleeds. One required ambulance admission. “ I needed 3 blood transfusions and 2 intravenous iron treatments because of Apixaban.” ‡ Discontinued 5 months before review and nosebleeds had settled.	6
1	#8	F	31	VTE	1	Apixaban #4	“No problems”	2
2	#9	M	53	AF	1	Rivaroxaban #6 [c]	Intolerable nosebleeds - worsened to a degree never experienced before. Needed intravenous iron and blood transfusions	6
2	#10	M	56	VTE	1	Rivaroxaban #7	Tolerable - not necessary to stop treatment	3
3	#11	F	55	VTE	1	Apixaban #5 [c]	Got worse, needed a bilateral Young’s Procedure (nostril closure [29])	3
2	#12	M	67	AF	1	Apixaban #6 [c]	Intolerable nosebleeds - worsened to a degree never experienced before. Needed intravenous iron and blood transfusions.	6
4	#13	M	56	VTE	1	Warfarin	Nosebleeds worse	w
					2	Rivaroxaban #8 [c]	Worsening, led to referral	3
4	#14	F	64	VTE	1	Rivaroxaban #9	No variation	2
5	#15	M	84	AF	1	Rivaroxaban #12	GI bleeding and epistaxis got worse (GI bleeding and massive epistaxis with need for transfusion) --> stopped NOAC	5
1	#16	F	62	VTE	1	Rivaroxaban #10	“Nosebleed would not stop for 2-3 hs - needed a 4 unit transfusion”	6
					2	Warfarin	Nosebleeds worse	w
					3	Heparin	No effect on nosebleeds	H

…/ctd

Table S1: Details of individual patients and treatments

…/ctd

HCP	Case	Gender	Age	Indication	Episode	Agent	Comment	Scale
1	#17	F	65	VTE	1	Warfarin	Tolerated with no ill effect, until severe gastrointestinal bleed	
					2	Apixaban #7	Tolerated with no ill effect	2
1	#18	F	57	VTE	2	Dabigatran #1	4-5 nosebleeds per day, but considered not really different to usual worst	3
6	#19	F	77	AF	1	Apixaban #8 [c]	Epistaxis were worse (ESS 4.75) compared to no anticoagulation	5
					2	Dabigatran #2	ESS reduced compared to Apixaban 0.51	2
6	#20	M	80	AF		Apixaban #9	Slight tolerable increase of epistaxis (ESS 0.51 to 1.92 after 6 weeks being on medication)	3
6	#21	F	62	VTE	1	Apixaban #10	ESS increased from 3.84 (daily, 6-15mins, not gushing) to 7.8 (several per day, gushing & anaemia)	3
3	#22	F	75	VTE	1	Apixaban #12	epistaxis worsen --> blood transfusions, laser treatment, nasal ointment, patient was treated to use nasal self-packing	3
3	#23	M	47	VTE	1	Apixaban #13	hardly any epistaxis, and no change	2
7	#24	F	71	VTE	1	Rivaroxaban #11	Epistaxis a little worse but really no problem	3
7	#25	F	65	AF		Apixaban #11	needed to reduce Apixaban to 2.5mg bd	4
7	#26	M	66	AF	1	Warfarin	Not tolerated- excessive epistaxis	w
					2	Dabigatran #3	Epistaxis improved	1
5	#27	M	71	AF	1	Apixaban #14	Apixaban well tolerated for almost 2 years with daily but tolerable nosebleeds	3
1	#28	M	54	AF	1	Warfarin	Fine, no bleeding issues, completed course	w
					2	Rivaroxaban #13	No change in nosebleeds- fine	2
			57	AF	3	Apixaban #15	"1-2hr gushers 2-3x per week then worst nosebleed ever- 10hs spurter. Scared, stopped and called hospital "	6
					4	Rivaroxaban #14	Bad, but not as bad as Apixaban: 1-2hr gushers 2-3x per week	3

Comments from patients"….", or clinicians' notes . [a]Patient advised to submit MHRA yellow card. [b]MHRA Yellow card submitted by patient. [c] Reason for referral to ERN centre.
