# Supplementary material for: Safety of direct oral anticoagulants in patients with hereditary hemorrhagic telangiectasia
Source: Orphanet J Rare Dis. 2019 Aug 28;14:210. doi: 10.1186/s13023-019-1179-1 (PMC6714298; doi:10.1186/s13023-019-1179-1)
Supplement: Supplementary file 2 — Table S2. Dosing regimes. (PDF 279 kb) [file 13023_2019_1179_MOESM2_ESM.pdf]

Safety of direct oral anticoagulants in patients with hereditary hemorrhagic telangiectasia

Shovlin CL, Millar CM, Droege F, Kjeldsen AD, Manfredi G, Suppressa P, Ugolini S, Coote N, Fialla AD, Geisthoff U, Lenato GM, Mager HJ, Pagella F,

Post MC, Sabbà C, Sure U, Torring PM, Dupuis-Girod S, Buscarini E, VASCERN-HHT.

Orphanet Journal of Rare Diseases 2019

ADDITIONAL FILE 2: Table S2: Dosing Regimes

Table S2: Dosing Regimes.

Rivaroxaban	Apixaban	Dabigatran
15 mg bd*<br>or 20mg od	10mg bd then<br>5mg bd*, or<br>2.5mg bd*	75 mg bd or<br>110mg bd, or<br>150mg bd [a]

All patients were treated with conventional dosing schedules and had normal renal function when subsequently evaluated by urea and electrolytes. [a] Dose reported to be "tolerated" by patient though experiencing nosebleeds 4-5 times a day. Note the doses of DOAC reported are those registered for patients with acute VTE (Rivaroxaban, Apixaban). In AF Rivaroxaban is dosed 20 mg o.d. (reduced to 15 mg with CrCl<50 mg/mL) and Apixaban 5 mg b.i.d. (reduced to 2.5 mg b.i.d with appropriate criteria). The doses of Dabigatran are those registered in patients with AF in USA.
