# Supplementary material for: Safety of direct oral anticoagulants in patients with hereditary hemorrhagic telangiectasia
Source: Orphanet J Rare Dis. 2019 Aug 28;14:210. doi: 10.1186/s13023-019-1179-1 (PMC6714298; doi:10.1186/s13023-019-1179-1)

Safety of direct oral anticoagulants in patients with hereditary hemorrhagic telangiectasia

Shovlin CL, Millar CM, Droege F, Kjeldsen AD, Manfredi G, Suppressa P, Ugolini S, Coote N, Fialla AD, Geisthoff U, Lenato GM, Mager HJ, Pagella F,

Post MC, Sabbà C, Sure U, Torring PM, Dupuis-Girod S, Buscarini E, VASCERN-HHT.

Orphanet Journal of Rare Diseases 2019

ADDITIONAL FILE 3:

Figure S1: Normal quantile plot of 4 point bleeding severity scale.

Figure S1: Normal quantile plot of 4 point bleeding severity scale.

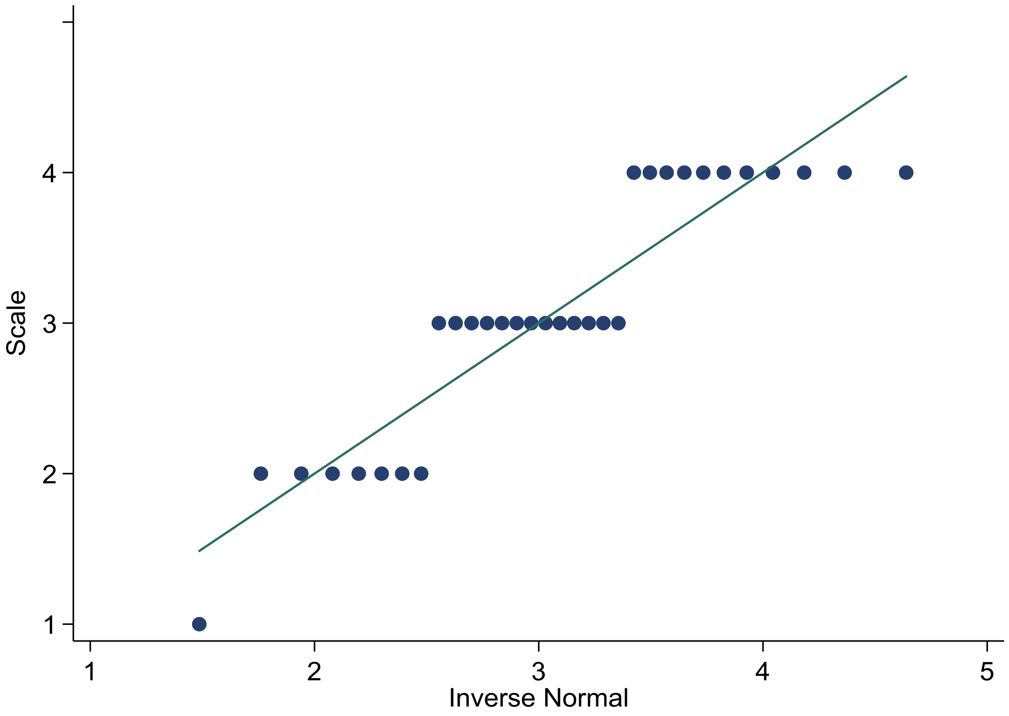

Supplement: Supplementary file 3 — Figure S1. Normal quantile plot of 4 point bleeding scale. (PDF 177 kb) [file 13023_2019_1179_MOESM3_ESM.pdf]
